# Investigation of Parameters that Affect the Success Rate of Microarray-Based Allele-Specific Hybridization Assays

**DOI:** 10.1371/journal.pone.0014777

**Published:** 2011-03-22

**Authors:** Lena Poulsen, Martin Jensen Søe, Lisbeth Birk Møller, Martin Dufva

**Affiliations:** 1 Department of Micro and Nanotechnology, DTU Nanotech, Technical University of Denmark, Lyngby, Denmark; 2 Department of Applied Human Molecular Genetics, Kennedy Center, Glostrup, Denmark; Ohio State University Medical Center, United States of America

## Abstract

**Background:**

The development of microarray-based genetic tests for diseases that are caused by known mutations is becoming increasingly important. The key obstacle to developing functional genotyping assays is that such mutations need to be genotyped regardless of their location in genomic regions. These regions include large variations in G+C content, and structural features like hairpins.

**Methods/Findings:**

We describe a rational, stable method for screening and combining assay conditions for the genetic analysis of 42 Phenylketonuria-associated mutations in the phenylalanine hydroxylase gene. The mutations are located in regions with large variations in G+C content (20–75%). Custom-made microarrays with different lengths of complementary probe sequences and spacers were hybridized with pooled PCR products of 12 exons from each of 38 individual patient DNA samples. The arrays were washed with eight buffers with different stringencies in a custom-made microfluidic system. The data were used to assess which parameters play significant roles in assay development.

**Conclusions:**

Several assay development methods found suitable probes and assay conditions for a functional test for all investigated mutation sites. Probe length, probe spacer length, and assay stringency sufficed as variable parameters in the search for a functional multiplex assay. We discuss the optimal assay development methods for several different scenarios.

## Introduction

Allele-specific oligonucleotide hybridization (ASH) on the massively parallel DNA microarray platform encompasses a simple and powerful method for high-throughput genotyping. ASH is widely used by Affymetrix as well as other companies, for analysis of single nucleotide polymorphisms (SNPs). As microarray technology is potentially a very powerful tool in diagnostics of known mutations causing diseases, we can expect an increase in the number of assay developers of clinical grade microarray-based assays. This paper aims to investigate the expected success rate of different strategies when developing ASH assays.

ASH exploits the decrease in stability of mismatch (MM) duplexes (e.g. mutant probe and wild-type target/allele) and perfect-match (PM) duplexes (e.g. wild-type probe and wild-type target/allele) when determining genotypes. Probes for ASH are usually designed so that the variant base/point mutation is situated centrally, because centrally placed mismatches have a higher destabilizing effect on the duplex than mismatches at the ends, and therefore give a better discrimination [1]–[5]. Discrimination is strongly dependant on probe length, and decreases dramatically with increases in probe length [3], [6]–[8]. Nevertheless, the use of very short probes [9] is generally not recommended, due to a lower signal yield of the capture probes [3] which compromises the sensitivity of the assay. Furthermore, the uniqueness of short probes declines with increasing complexity of the target [3]. One example of a highly complex target, is genomic DNA, whereas PCR products represent targets of lower complexity.

Probes can either be chosen experimentally or with the help of probe prediction algorithms or with a combination thereof. Probe prediction algorithms typically predict a “best fit” probe from certain input criteria, such as similar melting points (T*m*) or changes in Gibbs free energy of hybridization values, *ΔG*, and sequence uniqueness. *T*m and *ΔG* are equalized to allow discrimination at one assay condition, and uniqueness is required to avoid any unspecific hybridization. For genome-wide SNP analysis, the goal of the assay is to cover the genome with markers. In such cases difficult SNP loci can simply be avoided when designing the assay [10]–[12]. However, microarrays for diagnosis of disease-causing genetic mutations require a 100% assay success rate, suggesting that an alternative strategy for assay development is required.

We investigated the efficiency of a high-throughput probe characterization method for optimization of an allele-specific hybridization assay. As we tested crosswise a variety of probe lengths, assay stringencies and positions of probe sequence relative to the microarray surface, we covered all possible combinations of the most commonly modulated assay parameters. The data provide valuable information for DNA microarray assay developers.

## Materials and Methods

### DNA microarray probes

**Figure 1:**
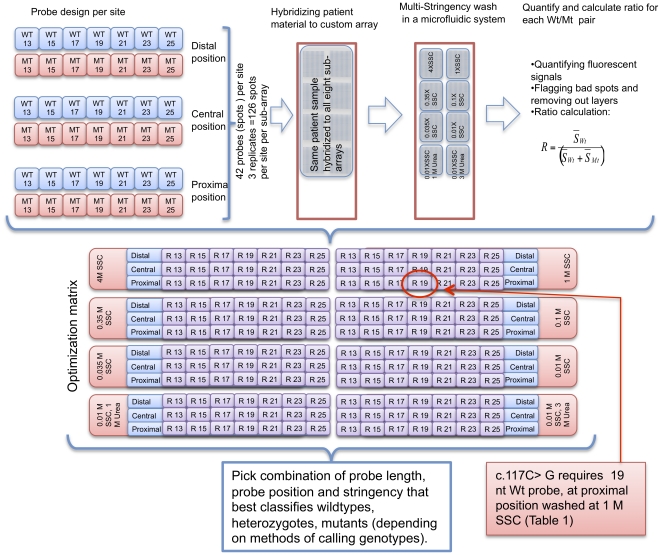
Experimental design. For each *PAH* mutation that was genotyped, 14 different probes were designed comprising 7 lengths (13 to 25-mer) wildtype (Wt) probe and corresponding *T*m-matched mutant (Mt) probe. For simplicity, Mt probes are denoted with the same number as corresponding Wt probe. In reality Mt probes could differ one or two bases in length in order to obtain similar calculated Tm as the corresponding mutant probe. The probes (*PAH* capture-sequence) were placed in three different positions: the proximal, central or distal part of the 60-mer oligonucleotide (Materials and Methods). With these triplicates of each probe there were a total of 42 spots/probes per *PAH* mutation. These 42 spots per SNP per probe position were repeated trice per subarray. The array was replicated in 8 identical sub-arrays on the custom-made Agilent slide. For mutations near a common SNP, additional probes were designed (see Materials and Methods). The microarray slide (all 8 sub-arrays) was hybridized with amplified patient material. The slide was then washed in a multi-stringency array washer. Each sub-array was washed at 37°C with different stringency wash buffers all containing 0.1% SDS and varying SSC (as indicated). Furthermore, the wash buffer for two sub-arrays contained the denaturant urea. After quantification (Materials and Methods) normalized ratios were calculated for each probe-pair. Alternative ratios (R_+2nt_ and R_−2nt_) (Supplementary Figure S1) were calculated as indicated. For R_+2nt_ a Wt probe (for example 15-mer) was combined with the Mt probe that was designed to *T*m-match the Wt probe that was 2 nucleotides longer (here, 17-mer Wt probe). The reverse was the case for the R_−2nt_. The probe-pair (length, position and ratio calculation) and stringency that resulted in the best classification genotypes near ideal values: wild-types 1, heterozygotes 0.5 and mutants 0 (Figure 3 and Table 1) are highlighted for three mutations (c.117C>G, c.143T>C and c.688G>A).

Wild-type (Wt) probes and mutant (Mt) probes were designed to target 42 different mutations in the human phenylalanine hydroxylase (*PAH*) gene (Figure 1 and Table 1). Probes were designed by using the sequence of the sense strand of *PAH*. For five mutations (c. 688G>A, c.727C>T, c. 730C>T, c. 734T>A and c. 1157A>G) in the proximity of common SNPs (696A>G, c. 735G>A or c. 1155G>C) probe-pairs were designed for both SNP alleles and for five mutations.

**Table 1:** Data for probes and assay conditions for the genotyping results shown in Figure 3.

*Number*	*Systematic name*	*Mutation name*	*Position*	*Match*	*Probe Length (Wt/Mt)*	*Calc. Tm (°C) Wt probe*	*Calc. Tm (°C) Mt probe*	*Calc ΔG (kcal/mol) Wt probe*	*Calc ΔG (kcal/mol) Mt probe*	*Stringency*
1	c.47_48delCT	L15/S16fsdelC orp.S16>XfsX1	Central	Mt-2	15/17	32.4	35.2	−12	−14	0.35 X SSC
2	c.60+5G>T	IVS1+5G>T	Proximal	Mt+2	19/18	55.1	53.3	−25	−22	4 X SSC
3	c.115_117delTTC	deltaF39/F39del	Central	Mt+2	15/13	39.0	31.9	−16	−14	0.35 X SSC
4	c.117C>G	F39L	Proximal	Tm	19/19	39.6	39.3	−17	−16	1 X SSC
5	c.136G>A	G46S	Distal	Tm	19/19	41.8	41.4	−17	−17	0.35 X SSC
6	c.140C>T	A47V	Proximal	Tm	25/24	49.4	48.7	−24	−21	0.35 X SSC
7	c.143T>C	L48S	Distal	Mt-2	17/18	43.8	46.3	−19	−21	0.35 X SSC
8	[c.187A>C;c.190C>A]	T63P/H64N	Central	Mt+2	19/17	41.3	39.9	−21	−19	1 M Urea
9	c.194T>C	I65T	Proximal	Mt+2	19/16	40.6	39.1	−19	−18	4 X SSC
10	c.204A>T	R68S	Proximal	Tm	15/14	34.9	34.6	−16	−14	1 X SSC
11	c.311C>A	A104D	Proximal	Mt+2	15/15	46.1	43.3	−17	−16	0.35 X SSC
12	c.329C>T	S110L	Proximal	Tm	15/17	37.1	37.3	−17	−17	4 X SSC
13	c.371C>T	T124I	Distal	Tm	17/17	31.4	31.6	−15	−14	0.35 X SSC
14	c.473G>A	R158Q	Proximal	Tm	19/21	45.8	45.6	−19	−20	0.1 X SSC
15	c.529G>C	V177L	Proximal	Mt-2	15/18	40.7	44.3	−17	−21	3 M Urea
16	c.663_664delAG	221/222 del AG/E221fs	Distal	Tm	15/15	26.3	25.8	−13	−12	4 X SSC
17	c.688G>A	V230I	Central	Mt-2	17/20	38.6	43.1	−16	−19	0.01 X SSC
18	c.727C>T-GG	R243X	Proximal	Tm	13/13	48.6	49.4	−21	−20	0.35 X SSC
19	c.727C>T-AA	R243X	Proximal	Tm	13/13	48.6	49.4	−21	−20	0.01 X SSC
20	c.730C>T-AG	P244S	Distal	Mt+2	15/13	51.5	40.3	−21	−15	0.35 X SSC
21	c.730C>T-GA	P244S	Proximal	Tm	21/22	64.8	65.8	−32	−32	3 M Urea
22	c.734T>A	V245E	Distal	Tm	19/19	62.6	62.2	−27	−28	1 M Urea
23	c.781C>T	R261X	Central	Mt+2	15/14	52.5	48.8	−21	−18	0.01 X SSC
24	c.782G>A	R261Q	Distal	Tm	15/16	51.3	51.2	−20	−19	1 M Urea
25	c.814G>T	G272X	Central	Mt-2	23/26	53	53.9	−28	−28	3 M Urea
26	c.842C>T	P281L	Central	Tm	13/15	38.3	36.5	−15	−15	1 M Urea
27	c.842+4A>G	IVS7nt4a>g	Central	Tm	15/14	37.8	38.4	−14	−15	1 M Urea
28	c.844G>A	D282N	Distal	Mt+2	21/21	54.5	50.4	−22	−21	0.1 X SSC
29	c.898G>T	A300S	Central	Tm	13/14	46.5	45.4	−18	−18	0.35 X SSC
30	c.916A>G	I306V	Central	Mt-2	17/17	38.4	44.1	−17	−18	0.35 X SSC
31	c.997C>T	L333F	Proximal	Tm	15/15	45.5	44.6	−17	−16	4 X SSC
32	c.1006C>T	Q336X	Distal	Tm	17/18	35.0	35.0	−16	−16	4 X SSC
33	c.1042C>G	L348V	Distal	Tm	13/15	48.5	48.3	−17	−17	1 M Urea
34	c.1066-11G>A	IVS10-11G>A	Central	Mt+2	15/16	47.1	47.0	−20	−19	0.35 X SSC
35	c.1068C>G	Y356X	Proximal	Mt-2	23/25	54.4	56.5	−26	−27	1 M Urea
36	c.1139C>T	T380M	Central	Mt+2	17/17	42.1	48.8	−15	−19	0.1 X SSC
37	c.1157A>G	Y386C	Distal	Tm	15/14	43.7	43	−18	−16	0.1 X SSC
38	c.1169A>G	E390G	Central	Mt+2	21/17	42.5	43.8	−19	−17	0.35 X SSC
39	c.1208C>T	A403V	Central	Tm	17/18	41.7	42.1	−18	−17	0.35 X SSC
40	c.1222C>T	R408W	Central	Mt-2	13/16	47.8	45.9	−20	−20	4 X SSC
41	c.1223G>A	R408Q	Distal	Tm	13/14	48.3	48.4	−20	−20	0.1 X SSC
42	c.1241A>G	Y414C	Distal	Tm	21/19	50.9	52.2	−23	−23	1 M Urea
43	c.1243G>A	D415N	Distal	Tm	15/17	42.3	42.3	−20	−19	0.35 X SSC
44	c.1315+1G>A	IVS12+1G>A	Central	Tm	19/22	31.9	31.5	−13	−14	1 X SSC

**The 44** ***PAH*** **sites included in this study and optimal assay conditions for each investigated site.** Site (mutations) name and exon/intron position in the *PAH* gene and the combination of assay conditions for each site for the best genotyping ratio (plotted in Figure 3). Probe sequence data are given in Supplementary Table S1. “Match” refers to the length of the corresponding mutant probe relative to the wild-type probe in the probe-pair. “Position” refers to location of specific probe sequence where “Proximal” denotes placement of the specific part close to the surface, “Central” denotes placement of the specific part in the middle of the 60 nt DNA and “Distal” denotes placement of the specific part at the end of the 60 nt DNA.

There were different SNP genotypes (c.735G>A) in the proximity of two mutations c.727C>T and c.730C>T. Hence, as the probes would overlap both the SNP and the mutation, these mutations were analyzed as four mutations (c.727C>T-GG, c.727C>T-AA, c.730C>T-AG and c.730C>T-GA). This brought the total number of mutant *PAH* alleles to 44. Most mutations (n = 38) were single-base substitutions, two were two-base substitutions and four were small (1–3 nt) conserved deletions (Table 1). Seven lengths (13 to 25-mer) in 2 nt increments of Wt probes with three different spacer lengths were designed for each mutation (Figure 1). Each Mt probe was designed to be as closely *T*m-matched to its respective Wt probe as possible. Thus, the Mt probe could be as long as the Wt probe or longer/shorter. The calculations of *T*m and *ΔG* are described in [13]. The variation in the calculated *T*m of all probe-pairs in the probe-set was kept to a minimum (within 6°C).

As the Agilent microarrays had a default of 60-mer probes, the probe was divided into three sections: proximal (closest to the microarray surface), central (in the middle) and distal (furthest away from the surface). All probes contained a *PAH*-specific sequence (capture sequence) and a spacer/filler sequence, selected for not hybridizing to the *PAH* targets (unpublished results). Twenty-one different probe-pairs were designed for each mutation; they comprised seven different lengths of the wild-type (Wt) capture sequence, 13–25 nucleotides in steps of two nucleotides, and their *T*m-matched mutant (Mt) probes in three different positions relative to the microarray surface (proximal, central and distal) (Figure 1). The positioning of the *PAH* capture sequence relative to the microarray surface was obtained by varying the location of the spacer/filler sequence [13].

### Agilent DNA microarrays

For genotyping the 44 mutated *PAH* alleles (Table 1) we used custom made (eArray 4.5) high-density *in situ* synthesized Agilent expression microarrays in the 8x15K format (Agilent Technologies, Palo Alto, CA). There were triplicates of each probe in the eight identical sub-arrays (Figure 1).

### DNA samples and target preparation

Genomic DNA (gDNA) samples used in this study originated from 38 individuals who were compound heterozygous (*n* = 31) or homozygous (*n* = 7) for mutation/s in the *PAH* gene [13]. Each mutation was genotyped separately. Homozygous mutants were only available for six different mutations. Thus 44 different mutated alleles were analysed by the 38 hybridization reactions. The original molecular diagnosis was made by denaturing gradient gel electrophoresis (DGGE) [14] followed by direct DNA sequencing.

Target preparation of *PAH* exons 1–12 with flanking sequences was carried out in a two-step process, involving PCR amplification of gDNA with an incorporated T7 promoter sequence followed by T7 *in vitro* transcription as described in [13]. Hence, the amplified target was complementary RNA (cRNA).

### Hybridization and stringency washes

Hybridization and stringency washes were carried out as described in [13] and Figure 1. Details regarding the in-house multi-stringency array washer (MSAW) are provided in [13].

### Stripping procedure for hybridized slides

In order to reuse the slides, hybridized targets were stripped off by using a modified version of the protocol used by Hahnke et al [15] as described in [13]. We reused each microarray up to three times. Although stripping off hybridized targets is not recommended by the manufacturer, several protocols describe successful reuse of stripped microarrays with reproducible results [15]–[18].

### Detection, quantification and data analysis

The processed microarrays were visualized by fluorescent scanning and the resulting images were quantified as described in [13]. Each mutation was genotyped separately. For assigning genotypes, a normalized ratio was calculated for each Wt and Mt probe-pair in the separate stringency zones. The normalized ratio (R) was calculated by dividing the average signal from the Wt probe by the sum of the signals from the average Wt and average Mt probes (S_Wt_/(S_Wt_+S_Mt_)) (Figure 1). The normalized ratio is also termed “relative allele signal” (RAS) in Affymetrix software. Furthermore, ratios were calculated for each Wt probe in combination with the Mt probe *T*m-matched with a shorter or longer (−2 or +2 nucleotides) Wt probe (Supplementary Figure S1). The Agilent 8x15K array format was chosen for the experiment because it allowed a multi-parametric test of probes for genotyping (about 15,000 probes/sub-array) in combination with multi-stringency washes of the eight identical sub-arrays, thereby maximizing the data output, and making the experiment practically and economically feasible. With triplicates of each probe sequence the total number of analyzed probes in each sub-array was about 3000 (the remaining probes were not included in this study). Quantification of the eight sub-arrays of each microarray slide and 38 hybridized microarray slides resulted in approximately 910,000 data points.

### Methods for assigning genotypes

We tested four different methods for assigning genotypes in order to evaluate the success rate of different assay designs. The methods for assigning genotypes were used to determine whether a probe-pair resulted in successful separation of the three potential genotypes: homozygous wild-type (Wt/Wt), heterozygote (Wt/Mt) or homozygous mutant (Mt/Mt).

**Figure 2:**
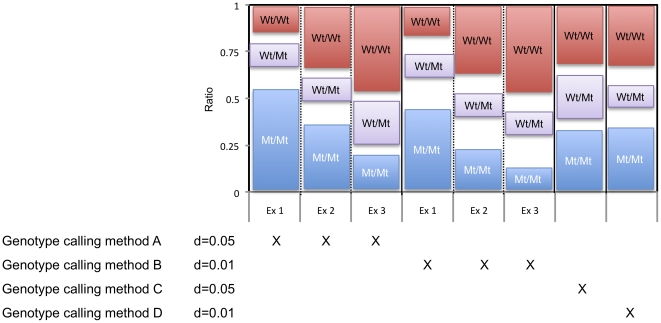
Methods A–D for calling genotypes. Graphical illustration of allowed ratio values for each method for calling genotypes. *Method A* requires that the difference (d) between the minimum Wt/Wt (wild-type) ratio and maximum Wt/Mt (heterozygote) ratio, as well as difference (d) between the minimum Wt/Mt (heterozygote) ratio and maximum Mt/Mt (mutant) ratio is >0.05. *Method B* requires that the difference (d) between the minimum Wt/Wt ratio and maximum Wt/Mt ratio, as well as the difference (d) between the minimum Wt/Mt ratio and maximum Mt/Mt ratio is >0.1. *Method C* requires that the minimum Wt/Wt ratio is >0.7, the maximum Wt/Mt ratio is <0.65, the minimum Wt/Mt ratio is >0.35 and the maximum Mt/Mt ratio is <0.3. *Method D* requires that the minimum Wt/Wt ratio is >0.7, the maximum Wt/Mt ratio is <0.6, the minimum Wt/Mt ratio is >0.4 and the maximum Mt/Mt ratio is <0.3. For methods A and B, three examples (Ex1–Ex3) of acceptable ratio values are shown. As the examples illustrate, the criteria for methods A and B for calling genotypes are fulfilled if the difference (d) between the different genotypes (wild-type, heterozygote and mutant) is greater than 0.05 or 0.1, respectively, regardless of whether the ratio values generally are high, intermediate or low.

Figure 2 depicts the four methods used to assign genotypes. Methods A and B only required separation of the normalized ratios of the three possible genotypes (wild-type, heterozygote and mutant) within a certain difference (d) between the minimum and maximum ratios observed for each respective genotype. Hence, genotyping was successful if the separation criteria were fulfilled, independent of whether wild-type, heterozygote and mutant ratios were high or low (Figure 2, examples 1–3). In contrast, methods C and D for assigning genotypes required that homozygous wild-types or mutants were less than 0.3 from their ideal ratio of 1 and 0, respectively, and heterozygotes less than 0.15 (method C) or 0.1 (method D) from their ideal ratio of 0.5. The distance “d” of 0.1 simulate a stronger test while and d = 0.05 a weaker test.

### Ethical considerations

The Kennedy Institute granted us permission to use archived anonymous samples from subjects investigated for mutations in the *PAH* gene. As these samples were only reanalysed for mutations that had already been diagnosed, we did not gain any additional genetic information about the subjects. Therefore there was no need to apply for an ethics approval or for further informed consent from the subjects.

## Results

For a multi-parametric test of probe choice strategies for genotyping with ASH-based assays, we combined custom made (Agilent) high-density microarrays with an in-house multi-stringency array washer which has been described in [13]. The modulated parameters were: the length of the *PAH* capture probe, the position of the *PAH* capture probe relative to the microarray surface, and finally the post-hybridization stringency wash. In order to make the experiment economically feasible, we reused each microarray up to three times by stripping the hybridized cRNA targets off with alkali and heat denaturation. Although the signal decreased with each successive re-hybridization, we were able to assign genotypes (data not shown). Control scans after the stripping procedure showed no signal from the microarray spots.

### Identification of performance-optimized assay for genotyping *PAH* mutations

**Figure 3:**
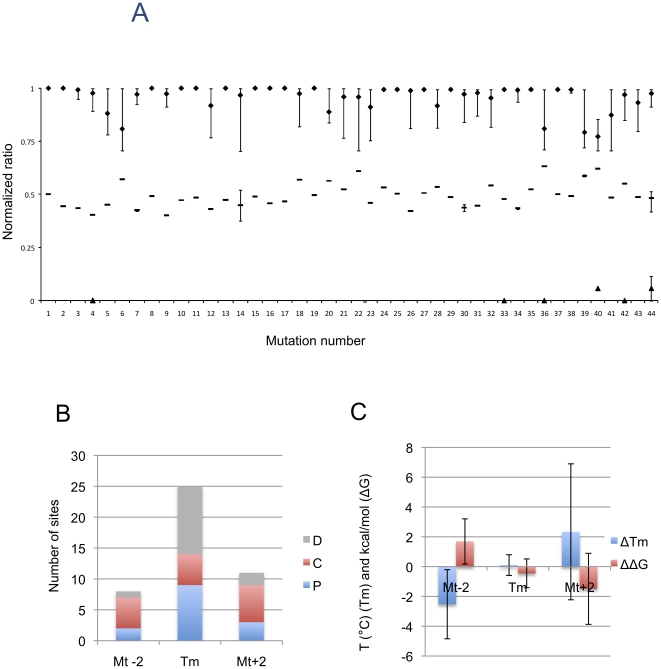
Genotyping the 44 different mutated *PAH* alleles*. A) For each mutation, the probe-pair (probe length, position from surface and Wt and Mt probe combination) and assay condition (stringency) for the best separation of genotypes is shown. Details about *PAH* mutations, probe-pairs and assay conditions are found in Table 1. Best separation was defined as wild-type (Wt/Wt) normalized ratios (see Materials and Methods section) clustering around 1, heterozygotes (Wt/Mt) around 0.5 and mutants (Mt/Mt) around 0. For each mutation, the average ratio of all samples carrying the wild-type DNA sequence on both alleles is represented by a diamond, the average normalized ratios for heterozygous samples is represented by a dash, and the average normalized ratio for homozygous mutated is represented by a triangle. Error bars show the observed minimum and maximum ratios. *42 unique *PAH* mutations and 44 different mutant *PAH* alleles were investigated (see Materials and Methods). B) Number of sites that were genotyped with Tm-matched probe-pairs or alternative probe-pairs The data was obtained by analysing Table 1. C) ΔTm (difference in Tm between wild type and mutant probe) and ΔΔG (difference between ΔG of wild type probe and mutant probe) of probe-pair function in the shown genotyped PAH mutations (A).

**Figure 4:**
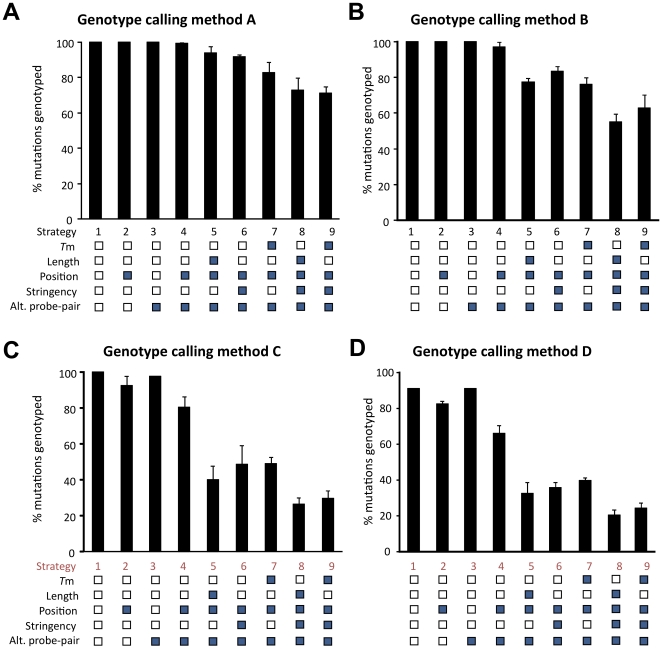
Percentage of successfully genotyped mutations for each assay strategy employed. Each column is the average percentage of successfully genotyped mutations obtained by using probes in the proximal, central and distal position (except for strategy 1 and 3 where all positions were varied). Error bars are maximum and minimum percentage of successfully genotyped mutations in one of the three positions (proximal, central or distal). A total of nine assay strategies (strategy 1–9) were investigated by varying (open square) or fixing (closed square) the following assay parameters: melting temperature *T*m, probe length, position, stringency and probe-pair combination. The performance of each assay strategy was found as the percentage of successfully genotyped mutations using **A**) Method A for calling genotypes, **B**) Method B for calling genotypes, **C**) Method C for calling genotypes and **D**) Method D for calling genotypes.

To illustrate the optimal separation of genotypes for each *PAH* mutation in this study, the best genotyping results relative to the ideal case were observed when wild-types gave ratios close to 1, heterozygotes close to 0.5 and (if available) homozygous mutated near 0 (Figure 3A). Table 1 shows the assay conditions that provide the optimal genotyping ratios for each mutation, *i.e.* combinations of probes, stringency, probe position and usages of non-Tm-matched probes. Some mutations were genotyped by many combinations of probes, stringency and probe positions, while others were only genotyped by one probe length and assay condition. As many combinations of probe length and assay conditions were tested, the identified probe lengths and assay conditions represent the *performance-optimized assay* for each respective site. Even with full freedom in the probe design and assay conditions, four mutations (c.473G>A, c.734T>A, c.1139C>T and c.1222C>T, or number 14, 22, 36 and 40, respectively) did not fulfil the criteria for method D for calling genotypes, but only fulfilled the remaining three methods for calling genotypes (A, B and C). This was due to the low heterozygote ratio (minimum ratio below 0.4 for c.473G>A) or high heterozygote ratio (above 0.6 for the remaining three mutations). However, it should be noted that other combinations of probe-pairs and assay stringency resulted in the successful assignment of genotypes, especially when using methods A and B (Figure 4).

The diversity of the assay parameters in the performance-optimized assay became apparent when analysing Table 1. The three possible probe positions were equally represented in the performance-optimized assay (distal: 14 sites, central 16 sites and proximal 14 sites) (Figure 3B). Furthermore, about 60% of the sites were genotyped with wild type and corresponding mutant probes that had the same calculated *T*m and *ΔG* (Figure 3B, Table 1). The remaining sites were genotyped with unbalanced probes with regard to *T*m and *ΔG* calculations. The unbalanced probe-pairs had 2 bases longer or shorter mutant probes when compared to the Wt probe. Unbalanced probe-pairs are referred to as “alternative probes” (Supplementary Figure S1). The probe-pairs with similar calculated *T*ms and *ΔGs* were over-represented at the proximal and distal positions in the performance-optimized assays, while the probe-pairs of alternative Mt probes were typically found in the central position (Figure 3B). As expected, the difference in calculated *T*m and *ΔG* between the wild-type and mutant probe in the performance-optimized probe-pairs (*ΔT*m and *ΔΔ*G respectively) showed that the *T*m-matched probe-pairs had *ΔT*ms close to zero with little spread. The *ΔΔ*Gs for *T*m-matched probe-pairs were also close to zero with little spread (Figure 3C). As expected, the alternative probes (probe-pairs) displayed a generally lower *ΔT*m for shorter mutant probes or higher *ΔT*m for longer mutant probes when compared to the *T*m-matched pairs. However, the average *ΔΔG* for pairs with alternative mutant probes was similar to that of the pairs that were *T*m-matched (Figure 3C). This was expected as the performance-optimized probes in a pair should have the same affinity in order to give heterozygote values close to 0.5. It should be noted that a large spread in *ΔT*m and *ΔΔ*G values was observed for the alternative probes-pairs (Figure 3C), indicating that we cannot fully explain ideal probe-pairs with thermodynamic calculations of the probe sequence alone.

### Assessment of the influence of optimization parameters on success rate when developing assays

Each method for calling genotypes (Figure 2) was analysed for the percentage of mutation sites that were successfully genotyped (success rate) by nine different assay strategies (Figure 4). The assay strategies had different combinations of fixed or flexible assay parameters, including assay stringency, probe design parameters and probe-pair combinations. For nearly all assay strategies, a decrease in success rate was observed in the direction from methods A and B for assigning genotypes to methods C and D. This was especially clear for assay strategies with limited flexibility, *e.g.* one probe length or *T*m-matched probe-set at one stringency, strategy 8 and 9, respectively. As expected, the highest success rates were observed when all or nearly all parameters were varied (Figure 4A–D). The three most flexible assay strategies (strategy 1–3, Figure 4) had 100% success-rate for methods A and B for calling genotypes. This implies that all 44 mutant alleles/42 unique mutations (see Materials and Methods) could be genotyped accurately and thus be implemented in a clinical assay. A 100% success-rate for method C was only obtained with full assay flexibility (strategy 1).

### Assay success rate when fixing calculated *T*m

The rationale of using *T*m-matching Wt and Mt probe-pairs was to obtain probes with similar melting points. Furthermore, when combining probe-pairs with similar *T*ms they should - in theory - function at common assay conditions. However, this strategy (strategy 9, Figure 4) resulted in one of the lowest success rates of the tested assay strategies, irrespective of which genotype assigning method was applied. Applying a stringency gradient alone, resulted in 10–20% higher success rates (compare strategy 7 and 9). This indicates that although probes were *T*m-matched within a *T*m range of 6°C they perform optimally at different stringencies, which supports previous observations [19]–[20].

### Assay success rate when fixing the probe length

Another assay strategy is simply to use one common probe length in the probe-set instead of *T*m-matching the entire probe-set. As previously mentioned, this study included seven different Wt probes, with lengths ranging from 13 to 25 nt, that were *T*m-matched with the respective Mt probes. The assay strategy with one common probe length and multiple stringencies produced a high success rate (above 90%) with method A for calling genotypes, but dropped below 40% when using the more stringent method D for assigning genotypes (Figure 4A and D, strategy 5). The assay strategy approach of choosing a fixed probe length resulted in success rates comparable to those for *T*m-matched probe-sets (compare strategy 8 and 9 in Figure 4). As with *T*m-matched probe-sets, processing microarrays at multiple stringencies resulted in higher success rates than when using a common stringency (compare strategy 5 and 8 in Figure 4). This is intuitive, as probes of the same length that target different mutation sites have different G+C contents, and hence, probes that require different stringencies in order to perform optimally. Therefore, a higher success rate was achieved by allowing a probe-set with probes of varying length and a *T*m to compensate for variances in the G+C content at the different mutation sites (strategy 6, Figure 4) as compared to the success rate when fixing these parameters (Figure 4, strategy 8 and 9).

### The influence of the capture-probe position relative to the microarray surface

When different spacer lengths were included as varying assay parameters, the assay success rate increased for methods C and D for assigning genotypes (Figure 4). A general analysis underlined that fixed probe positions (as in strategy 2 and 4) gave poorer assay success rates than those achieved by placing probes at different positions in the final assay (as in strategy 1 and 3). However, this was most pronounced when applying methods C and D and less when applying methods A and B (Figure 4).

### The effect of method-choice for assigning genotype on assay success rate

In a genotyping assay, the wildtype-to-mutant signal intensity ratio of individual genotypes (homozygous wildtypes, hetetozygotes and homozygous mutants) should preferably be clearly separated. As expected, the methods for assigning genotypes (Figure 2) that require the greatest (0.1) separation of genotypes, methods B and D, had an approximately 10% lower assay success rate, than methods A and C, respectively (Figure 4).

## Discussion

Allele-specific hybridization (ASH) to DNA microarrays is commonly used for SNP genotyping and mutation analysis. When setting up ASH-based genotyping assays some rules of thumb have been established to ensure the likelihood of obtaining a functional assay. Probes should be short (often 15–25 nucleotides), with the mismatch placed in the middle of the probe to maximize the signal difference obtained from mismatch and perfect-match hybrids. Probes with similar working optima are also chosen because microarrays are often processed under one particular assay condition (hybridization and stringency wash temperature and buffer composition). This is a challenge when addressing target sequences with varying G+C contents. Melting temperature (*T*m) calculations are based on thermodynamic models that use *solution parameters* and an assumption that reactions have reached equilibrium. Consequently, *T*m calculations do not fully predict hybridization and dissociation on microarray surfaces [2], [4], [21]. Only weak correlations have been observed between calculated *T*ms and the temperature at which optimal assay performance was obtained [19]–[20]. We solved these limitations by developing assays by systematically varying parameters that influence assay specificity, *i.e.* assay stringency, probe length and probe position relative to surfaces. After the initial screening of probes, the next step is to select a combination of probe length, probe position and assay stringency that fulfill the criteria for assigning a genotype for each mutation site. In fact our approach is the reverse of regular SNP assay development in which the assay conditions are fixed and the SNPs that function within the set conditions, are selected [10]–[12].

We observed that the method of choice for assigning genotypes, had a large impact on the fraction of successfully genotyped mutations with a given assay strategy (Figure 4). The success rate of methods A and B was higher for most assay strategies than that of methods C and D. The likely reason is that methods C and D necessitate probe ratios in the proximity of the ideal values for each genotype class (1 =  wild-type homozygous, 0.5 =  heterozygous and 0 =  homozygous mutant). In order to obtain ideal ratios, each probe-pair (mutant and wild-type probe) in a probe-set must have similar assay condition preferences and similar *ΔG* values in order to function in assay. In contrast, methods A and B only require that the Wt- and corresponding Mt-probe signals can be discriminated at an optimal stringency. Methods C and D are therefore very sensitive to the relative stability of perfect-match and the corresponding mismatch hybrid/duplex. Interestingly, by screening probes of different lengths, their position relative to the surface and assay washing conditions, it was possible to find probes and conditions where the Wt- and Mt-probes had similar stability and thus, a value of 0.5 for heterozygotes. As noted above (Figure 3C), the *ΔG* values between wild-type and corresponding mutant probes could vary significantly, indicating that we do not fully understand the formation of hybrids at the array surface. This further supports the fact that screening probes and conditions are warranted.

Assay strategies that employed multiple assay conditions (stringencies) were easier to obtained functional assays for than strategies that used one common optimized condition. This corroborates the findings of a smaller scale diagnostic assay using spotted microarrays of *T*m-matched probes immobilized on agarose coated slides [20], [22], as well as other studies [6], [13], [19], [23]–[29]. The effect of introducing restrictions in assay conditions appears to be cumulative and the more restrictions introduced the less the likelihood of obtaining a functional assay (Figure 4). The commonly used assay strategy 9, *i.e.* fixed *T*m, probe position and stringency, only results in a 20–70% success rate depending on the method used for assigning genotypes (Figure 4). In contrast, allowing the variation of all parameters at the same time will give assays that successfully genotype 100% of the mutation sites.

We have previously shown that each part of the 60 nt probe exposes the respective hybrid to different stringencies [13]. Stringency at the distal position of the polymer was significantly less than at the central and proximal positions which explains the more solution-like properties of probes placed on longer spacers [13], [30]–[33]. This suggests that probes should be placed distally. However, in the performance-optimized assay described in Table 1 and Figure 3A, only a third of the sites were genotyped with probes placed distally (Figure 3B) indicating that both proximal and central positions create a nano-environment that is superior to that found at the distal end. The mechanism is still unclear, but we speculate that conformations in targets and probes as well as the length of the tail of the target might play a role in creating unique nano-environments. Finding computer models for these interactions is not the scope of this article, but we demonstrate that a functional assay can be achieved with sufficient probe/assay screening.

**Figure 5:**
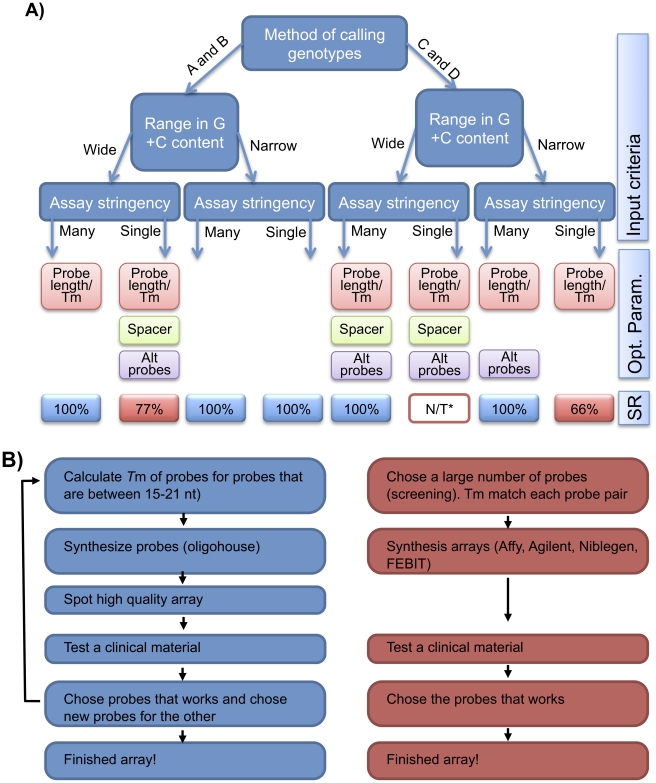
Overview over different assay development strategies. **A**) After choosing the method for calling genotypes (Figure 2) or for genotyping loci, placed in genomic regions with wide to narrow ranges in G+C content, the choice to run the assay at a single or at many assay stringencies is made. Based on the above selections “Input criteria” the parameters (Opt. Param.) that need to be optimized/flexible are shown below with the achieved success rate (SR) (percentage of mutations successfully genotyped). The parameters that must be optimized are probe length/*T*m, spacer length (position of probe relative to array surface) and alternative combinations of wildtype and mutant probe in probe-pairs (Alt probes). The success rate obtained in this study (genotyping of *PAH* mutations) is valid for the wide range in G+C content. The results from a narrow range in G+C content are from genotyping mutations in the *HBB* gene (reference). **B**) The steps in bottom-up (left) and top-down (right) assay strategies are listed. The bottom-up approach is an iterative process with many rounds of probe design, testing and the redesigning of probes. In contrast the top-down approach only utilizes one optimization experiment including all parameters needed for a functional assay.

In conclusion, our experiments showed that the selected method for assigning genotypes if the assays are run with or without varying stringency had a great impact on the success rate of the different assay strategies. We therefore recommend a top-down strategy (Figure 5), when developing an ASH-based genotyping assay. This implies an initial selection of method for assigning genotypes. Secondly, it is important to consider if the mutations/SNP lies in areas with high or low variance in GC content variance. Thirdly it is important to determine if the final assay is to be performed at one or many stringencies. From these input criteria an assay optimization procedure can be chosen to obtain a final assay where all the desired mutations/SNP's can be analysed (Figure 5A). *PAH* genotyping was tested with all four genotype-calling methods. Mutations in the *PAH* gene lies in region with highly varying GC content. Obtaining a final assay genotyping mutations in the PAH genes required different optimization procedure depending mainly on the method to call genotypes. Methods A and B which accepts non-ideal probe behaviour (see above) requires that the assay was optimized using assay parameters stringency and probe length. In contrast genotype-calling methods C and D that accepts only ideal probe behaviour required that all four parameters were optimized (probe length/Tm, spacer, alternative probes and stringency).

In contrast to the *PAH* genotyping assay in this study, assays targeting genomic regions with a narrower span in G+C content require optimization of fewer parameters. Previous papers regarding identification of optimal assay conditions for genotyping mutations in the *HBB* gene found that an optimization only involved probe length and stringency [20], [22] (Figure 5). This is probably due to the relatively few (nine) *HBB* genotyped mutations and that the mutations were placed in genomic regions with a lower span (45–75%) in G+C content in contrast to the mutations in the *PAH* gene (20–75%).

Assay optimization can be performed along two routes (Figure 5B). An iterative trial and error process can be employed when limited optimization is needed (Figure 5B left panel). For example, the iterative process is successful when optimizing the assay stringency and probe set for a small set of mutations [22, 34]. Alternatively, as shown here, a non-iterative process can be employed (Figure 5B right panel). This latter optimization strategy can save time, valuable patient material and most likely costs, as all essential parameters influencing assay functionality are tested in one experiment. One drawback of this optimization strategy with high-density microarrays, is that the optimized probe-set needs to stay on the same or similar microarray platform, e.g. Agilents microarrays. Because the same probe-set cannot be expected to function on a spotted oligonucleotide array on a different microarray substrate, or will at least require further optimisation or validation.

## Supporting Information

Figure S1 Experimental strategy, use of alternative probes.
(3.17 MB TIF)

Table S1 (0.06 MB PDF)
