# Supplementary figures and images for: Investigation of Parameters that Affect the Success Rate of Microarray-Based Allele-Specific Hybridization Assays
Source: PLoS One. 2011 Mar 22;6(3):e14777. doi: 10.1371/journal.pone.0014777 (PMC3062541; doi:10.1371/journal.pone.0014777)

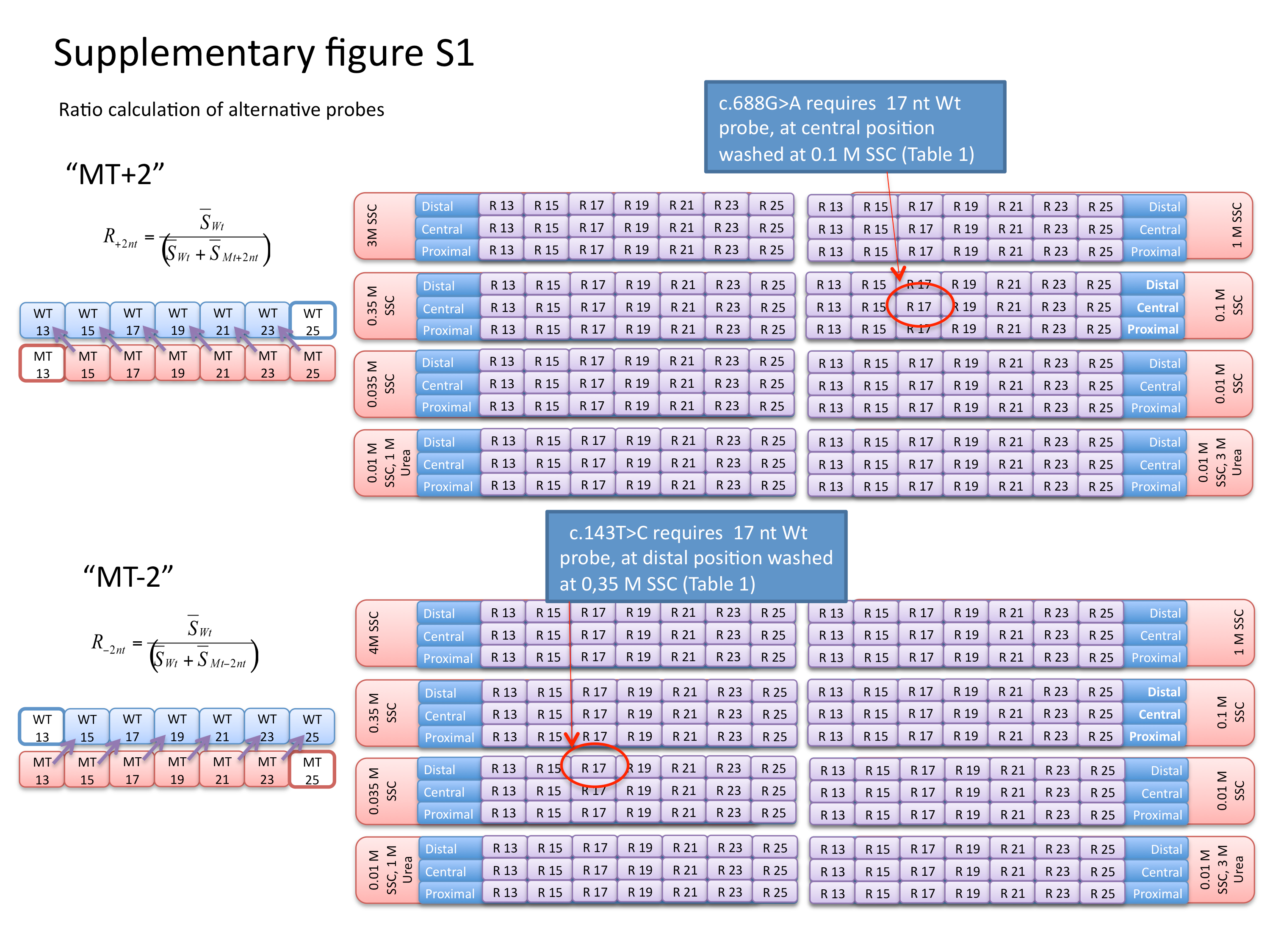

Supplement: Figure S1 — Experimental strategy, use of alternative probes. (3.17 MB TIF) [file pone.0014777.s002.tif]
