# Supplementary material for: Investigation of Parameters that Affect the Success Rate of Microarray-Based Allele-Specific Hybridization Assays
Source: PLoS One. 2011 Mar 22;6(3):e14777. doi: 10.1371/journal.pone.0014777 (PMC3062541; doi:10.1371/journal.pone.0014777)
Supplement: Table S1 — (0.06 MB PDF) [file pone.0014777.s001.pdf]

Supplementary table 1

Index no	Systematic name	Mutation name	Position	Type	Match	Probe Length	Sequence	Calculated Tm	DTM	Calc DG	DDG	Stringency
1	c.47_48delCT	L15/S16fsdelCorp.S16>XfsX1	Central	Wt	Mt-2	15	AACTCTCTGACTTTG	32.4	-2.8	-12	2.7	0.35 X SSC
				Mt		17	GAAACTCTGACTTTGGA	35.2		-14		
2	c.60+5G>T	IVS1+5G>T	Proximal	Wt	Mt+2	19	GACAGgtgagccacggcag	55.1	1.8	-25	-2.3	4 X SSC
				Mt		18	GGACAGgtgatccacggc	53.3		-22		
3	c.115_117delTTC	deltaF39/ F39del	Central	Wt	Mt+2	15	CTGATCTTCTCACTC	39.0	7.1	-16	-2.0	0.35 X SSC
				Mt		13	ACTGATCTCACTC	31.9		-14		
4	c.117C>G	F39L	Proximal	Wt	Tm	19	ACTGATCTTCTCACTCAAA	39.6	0.3	-17	-1.3	1 X SSC
				Mt		19	ACTGATCTTGTCACTCAAA	39.3		-16		
5	c.136G>A	G46S	Distal	Wt	Tm	19	GAAGAAGTTGGTGCATTGG	41.8	0.4	-17	0.2	0.35 X SSC
				Mt		19	AAGAAGTTAGTGCATTGGC	41.4		-17		
6	c.140C>T	A47V	Proximal	Wt	Tm	25	AAGAAGTTGGTGCATTGGCCAAAGT	49.4	0.7	-24	-2.6	0.35 X SSC
				Mt		24	AGAAGTTGGTGTATTGGCCAAAGT	48.7		-21		
7	c.143T>C	L48S	Distal	Wt	Mt-2	17	TGGTGCATTGGCCAAAG	43.8	-2.5	-19	1.8	0.35 X SSC
				Mt		18	TTGGTGCATCGGCCAAAG	46.3		-21		
8	[c.187A>C;c.190C>A]	T63P/H64N	Central	Wt	Mt+2	19	TAAACCTGACCCACATTGA	41.3	1.4	-21	-1.9	1 M Urea
				Mt		17	AAACCTGCCCAACATTG	39.9		-19		
9	c.194T>C	I65T	Proximal	Wt	Mt+2	19	TGACCCACATTGAATCTAG	40.6	1.5	-19	-0.9	4 X SSC
				Mt		16	ACCCACACTGAATCTA	39.1		-18		
10	c.204A>T	R68S	Proximal	Wt	Tm	15	AATCTAGACCTTCTC	34.9	0.3	-16	-1.9	1 X SSC
				Mt		14	AATCTAGTCCTTCT	34.6		-14		
11	c.311C>A	A104D	Proximal	Wt	Mt+2	15	ATTGGTGCCACTGTC	46.1	2.8	-17	-0.5	0.35 X SSC
				Mt		15	TTGGTGACACTGTCC	43.3		-16		
12	c.329C>T	S110L	Proximal	Wt	Tm	15	GAGCTTTCACGAGAT	37.1	-0.2	-17	-0.2	4 X SSC
				Mt		17	CAGAGCTTTTACGAGAT	37.3		-17		
13	c.371C>T	T124I	Distal	Wt	Tm	17	CCAAGAACCATTCAA	31.4	-0.2	-15	-1.3	0.35 X SSC
				Mt		17	CCAAGAATCATTCAAGA	31.6		-14		
14	c.473G>A	R158Q	Proximal	Wt	Tm	19	GTGCAAGACGGAAGCAGTT	45.8	0.2	-19	0.4	0.1 X SSC
				Mt		21	CGTGCAAGACAGAAGCAGTTT	45.6		-20		
15	c.529G>C	V177L	Proximal	Wt	Mt-2	15	CCCTCGAGTGGAATA	40.7	-3.6	-17	3.8	3 M Urea
				Mt		18	CATCCCTCGACTGGAATA	44.3		-21		
16	c.663_664delAG	221/222 del AG/ E221fs	Distal	Wt	Tm	15	CCATGAAGATAACAT	26.3	0.5	-13	-0.7	4 X SSC
				Mt		15	TCCATGAATAACATT	25.8		-12		
17	c.688G>A	V230I	Central	Wt	Mt-2	17	TGGAAGACGTTTCTCAg	38.6	-4.5	-16	3.1	0.01 X SSC
				Mt		20	CTGGAAGACATTTCTCAgTT	43.1		-19		
18	c.727C>T-GG	R243X	Proximal	Wt	Tm	13	CGCCTCCGACCTG	48.6	-0.8	-21	-0.4	0.35 X SSC
				Mt		13	TCCGCCTCTGACC	49.4		-20		
19	c.727C>T-AA	R243X	Proximal	Wt	Tm	13	CGCCTCCGACCTG	48.6	-0.8	-21	-0.4	0.01 X SSC
				Mt		13	TCCGCCTCTGACC	49.4		-20		
20	c.730C>T-AG	P244S	Distal	Wt	Mt+2	15	CCTCCGACCTGTaGC	51.5	11.2	-21	-6.0	0.35 X SSC
				Mt		13	CCTCCGATCTGTa	40.3		-15		
21	c.730C>T-GA	P244S	Proximal	Wt	Tm	21	CCGCCTCCGACCTGTgGCTGG	64.8	-1	-32	0.1	3 M Urea
				Mt		22	CCGCCTCCGATCTGTgGCTGGC	65.8		-32		
22	c.734T>A	V245E	Distal	Wt	Tm	19	TCCGACCTGTgGCTGGCCT	62.6	0.4	-27	1.6	1 M Urea
				Mt		19	CCGACCTGCgGCTGGCCTG	62.2		-28		
23	c.781C>T	R261X	Central	Wt	Mt+2	15	GGCCTTCCGAGTCTT	52.5	3.7	-21	-2.6	0.01 X SSC
				Mt		14	GGCCTTCTGAGTCT	48.8		-18		
24	c.782G>A	R261Q	Distal	Wt	Tm	15	GCCTTCCGAGTCTTC	51.3	0.1	-20	-0.9	1 M Urea
				Mt		16	GGCCTTCCAAGTCTTC	51.2		-19		
25	c.814G>T	G272X	Central	Wt	Mt-2	23	ACATCAGACATGGATCCAAGCCC	53	-0.9	-28	-0.6	3 M Urea
				Mt		26	GTACATCAGACATTGATCCAAGCCCA	53.9		-28		
26	c.842C>T	P281L	Central	Wt	Tm	13	CCGAACCgtgagt	38.3	1.8	-15	-0.2	1 M Urea
				Mt		15	CCGAACTgtgagtac	36.5		-15		
27	c.842+4A>G	IVS7nt4a>g	Central	Wt	Tm	15	AACCgtgagtactgt	37.8	-0.6	-14	0.6	1 M Urea
				Mt		14	AACCgtgggtactg	38.4		-15		
28	c.844G>A	D282N	Distal	Wt	Mt+2	21	gtctttcagTGACATCTGCCA	54.5	4.1	-22	-1.6	0.1 X SSC
				Mt		21	gtctttcagTAACATCTGCCA	50.4		-21		
29	c.898G>T	A300S	Central	Wt	Tm	13	AGCTTTGCCCAGT	46.5	1.1	-18	0.0	0.35 X SSC
				Mt		14	CAGCTTTTCCCAGT	45.4		-18		
30	c.916A>G	I306V	Central	Wt	Mt-2	17	tacagGAAATTGGCCTT	38.4	-5.7	-17	1.3	0.35 X SSC
				Mt		17	tacagGAAGTTGGCCTT	44.1		-18		
31	c.997C>T	L333F	Proximal	Wt	Tm	15	GTTTGGGCTCTGCAA	45.5	0.9	-17	-1.3	4 X SSC
				Mt		15	AGTTTGGGTTCTGCA	44.6		-16		
32	c.1006C>T	Q336X	Distal	Wt	Tm	17	TCTGCAAACAAGGAGAC	35.0	0	-16	-0.2	4 X SSC
				Mt		18	TCTGCAAATAAGGAGACT	35.0		-16		
33	c.1042C>G	L348V	Distal	Wt	Tm	13	GGGCTCCTGTCAT	48.5	0.2	-17	-0.4	1 M Urea
				Mt		15	TGGGCTCGTGTCATC	48.3		-17		
34	c.1066-11G>A	IVS10-11G>A	Central	Wt	Mt+2	15	ttcacttggggccta	47.1	0.1	-20	-0.6	0.35 X SSC
				Mt		16	tcacttagggcctaca	47.0		-19		
35	c.1068C>G	Y356X	Proximal	Wt	Mt-2	23	ggcctacagTACTGCTTATCAGA	54.4	-2.1	-26	1.5	1 M Urea
				Mt		25	ggcctacagTAGTGCTTATCAGAGA	56.5		-27		
36	c.1139C>T	T380M	Central	Wt	Mt+2	17	CACTGTCATGGAGTTC	42.1	-6.7	-15	4.1	0.1 X SSC
				Mt		17	CACTGTCACGGAGTTCC	48.8		-19		
37	c.1157A>G	Y386C	Distal	Wt	Tm	15	CCCCTcTATTACGTG	43.7	0.7	-18	-2.3	0.1 X SSC
				Mt		14	CCCTcTGTTACGTG	43		-16		
38	c.1169A>G	F390G	Central	Wt	Mt+2	21	TACGTGGCAGAGAGTTTTAAT	42.5	-1.3	-19	-2.2	0.35 X SSC

Supplementary table 1

[illegible]	[illegible]	[illegible]	[illegible]	Mt		17	GTGGCAGGGAGTTTTAA	43.8		-17	[illegible]	[illegible]
39	c.1208C>T	A403V	Central	Wt	Tm	17	GAACTTTGCTGCCACAA	41.7	-0.4	-18	-0.8	0.35 X SSC
				Mt		18	gGAACTTTGTTGCCACAA	42.1		-17		
40	c.1222C>T	R408W	Central	Wt	Mt-2	13	ATACCTCGGCCCT	47.8	1.9	-20	-0.1	4 X SSC
				Mt		16	CAATACCTTGGCCCTT	45.9		-20		
41	c.1223G>A	R408Q	Distal	Wt	Tm	13	TACCTCGGCCCTT	48.3	-0.1	-20	0.3	0.1 X SSC
				Mt		14	TACCTCAGCCCTTC	48.4		-20		
42	c.1241A>G	Y414C	Distal	Wt	Tm	21	TCAGTTCGCTACGACCCATAC	50.9	-1.3	-23	0.3	1 M Urea
				Mt		19	AGTTCGCTGCGACCCATAC	52.2		-23		
43	c.1243G>A	D415N	Distal	Wt	Tm	15	TCGCTACGACCCATA	42.3	0	-20	-0.3	0.35 X SSC
				Mt		17	TCGCTACAACCCATACA	42.3		-19		
44	c.1315+1G>A	IVS12+1G>A	Central	Wt	Tm	19	CCATTAACAgtaagtaatt	31.9	0.4	-13	0.5	1 X SSC
				Mt		22	TCCATTAACAataagtaattta	31.5		-14		

References

1. Nowacki et al. Nucleic Acid Res. 1997, 25(1) 139-142
2. Hoang et al. Nucleic Acid Res. 1996, 24(1) 127-131
3. Guldberg et al. Am J Hum Genet. 1998, 63(1) 71-79
